# Molecular Typing of Lung Adenocarcinoma on Cytological Samples Using a Multigene Next Generation Sequencing Panel

**DOI:** 10.1371/journal.pone.0080478

**Published:** 2013-11-13

**Authors:** Aldo Scarpa, Katarzyna Sikora, Matteo Fassan, Anna Maria Rachiglio, Rocco Cappellesso, Davide Antonello, Eliana Amato, Andrea Mafficini, Matilde Lambiase, Claudia Esposito, Emilio Bria, Francesca Simonato, Maria Scardoni, Giona Turri, Marco Chilosi, Giampaolo Tortora, Ambrogio Fassina, Nicola Normanno

**Affiliations:** 1 ARC-NET Research Centre, University of Verona, Verona, Italy; 2 Department of Pathology and Diagnostics, University of Verona, Verona, Italy; 3 Pharmacogenomic Laboratory, INT-Fondazione Pascale, Centro di Ricerche Oncologiche di Mercogliano, Mercogliano, Italy; 4 Department of Medicine, University of Padova, Padova, Italy; 5 Department of Medicine, Oncology Unit, University of Verona, Verona, Italy; 6 Cell Biology and Biotherapy Unit, INT-Fondazione Pascale, Napoli, Italy; UNIVERSITY MAGNA GRAECIA, Italy

## Abstract

Identification of driver mutations in lung adenocarcinoma has led to development of targeted agents that are already approved for clinical use or are in clinical trials. Therefore, the number of biomarkers that will be needed to assess is expected to rapidly increase. This calls for the implementation of methods probing the mutational status of multiple genes for inoperable cases, for which limited cytological or bioptic material is available. Cytology specimens from 38 lung adenocarcinomas were subjected to the simultaneous assessment of 504 mutational hotspots of 22 lung cancer-associated genes using 10 nanograms of DNA and Ion Torrent PGM next-generation sequencing. Thirty-six cases were successfully sequenced (95%). In 24/36 cases (67%) at least one mutated gene was observed, including *EGFR, KRAS, PIK3CA, BRAF, TP53, PTEN, MET, SMAD4, FGFR3, STK11, MAP2K1*. *EGFR* and *KRAS* mutations, respectively found in 6/36 (16%) and 10/36 (28%) cases, were mutually exclusive. Nine samples (25%) showed concurrent alterations in different genes. The next-generation sequencing test used is superior to current standard methodologies, as it interrogates multiple genes and requires limited amounts of DNA. Its applicability to routine cytology samples might allow a significant increase in the fraction of lung cancer patients eligible for personalized therapy.

## Introduction

Lung cancer is the leading cause of cancer-related death worldwide [1], [2], [3]. It is classified as small cell or non-small cell lung cancer (NSCLC), the latter comprising three of the most common subtypes: adenocarcinoma, squamous cell carcinoma, and neuroendocrine tumors [4].

The majority of NSCLC are diagnosed at an advanced stage with inoperable disease [5]. Therefore, in more than 85% NSCLC minimally invasive procedures must be employed to obtain diagnostic material, which is consequently represented by either small biopsies or cytology samples [5], [6]. This significantly affects the morphological and molecular characterization required for targeted therapies, whose efficacy is limited to patients with specific genetic alterations [5].

For lung adenocarcinomas, epidermal growth factor receptor (EGFR) tyrosine kinase inhibitors have been approved for treatment of tumors carrying *EGFR* gene mutations, and crizotinib for tumors with anaplastic lymphoma kinase (*ALK*) gene rearrangements [7], [8], [9], [10]. Clinical trials are ongoing in subgroups of patients harboring specific molecular alterations such as *BRAF*, *PIK3CA* or *KRAS* activating mutations [11], [12], [13]. Therefore, the number of predictive biomarkers to be assessed for novel targeted drugs entering into clinical practice is expected to rapidly increase [2], [10], [14].

Sanger sequencing is currently the most widely applied technique in the characterization of *EGFR* gene status in clinical practice [15]. Real-time PCR-based methods have been shown to efficiently detect *EGFR* mutations in samples containing 1% mutated cancer cells [16]. However, there is no sufficient information on the predictive ability of these techniques, since no clear correlation has been established up to now between the quantity of mutant alleles in the cancer and the extent and duration of response to therapy [16], [17]. More importantly, most methods have been developed and validated to assess single gene alterations. Massive parallel sequencing, also known as next generation sequencing (NGS) or deep sequencing, has been recently introduced and is the most sensitive approach to index multiple genes starting from a limited amount of DNA [18].

The Ion AmpliSeq Colon and Lung Cancer Panel (Lifetechnologies, Carlsbad, CA, USA) multigene next generation sequencing (NGS) allows assessment in a single analysis of hotspot mutations in 22 genes related to lung and colon tumorigenesis. The panel has been validated though a collaborative effort of 8 European institutions (http://tools.invitrogen.com/content/sfs/brochures/AmpliSeq-Colon-Lung-Cancer-Panel-Flyer.pdf).

With the present study, the performance of the Ion AmpliSeq Colon and Lung Cancer Panel was investigated in a series of lung adenocarcinoma cytological samples to define its diagnostic relevance.

## Materials and Methods

### Ethic statement

Written informed consent was obtained from all patients involved in the study, which was approved in the final form by the Ethics Committee of the Azienda Ospedaliera e Università degli Studi di Padova (N. 0002537 in January 16th, 2013). All the samples were received anonymously and processed at the Molecular Pathology Unit of the Department of Pathology and Diagnostics at the University of Verona.

### Samples

A series of 38 lung adenocarcinoma trans-thoracic fine needle aspiration (FNA) cytology specimens consecutively collected in 2012 at the Surgical Pathology and Cytopathology Unit of Padua University and the Pharmacogenomic Laboratory of the INT-Fondazione Pascale in Napoli, were studied (**Table 1**). In two cases a matched tumor biopsy was also available. The original routine slides were re-assessed by three pathologists (AS, MF and AF) according to current WHO criteria [4].

**Table 1:** Clinico-pathological features of the considered series.

Characteristic		#
Gender	Male	24 (63.2%)
	Female	14 (36.8%)
Age	-	69±9 (median 68; range 48–85)
	G1	7 (18.4%)
Grading	G2	27 (71.1%)
	G3	4 (10.5%)
	Stage IIIA	7 (18.4%)
Staging	Stage IIIB	4 (10.5%)
	Stage IV	11 (28.9%)
	missing	16 (42.1%)
Sources	Cytologial smears	21 (55.3%)
	FNA whasings	17 (44.7%)

The series included 21 cytological smears and 17 fine needle aspirate (FNA) washings:

- Routine smear cytological slides fixed with Cytofix® (Bio-Fix 05-x200®, Bio-Optica, Milano, Italy) and stained with Papanicolaou or Diff-Quick. Tumor cells were scraped from one original smear, by manually microdissection at the microscope in order to obtain at least 100 tumor cells. A mean number of 1,050±1,480 tumor cells per slide were retrieved (range 100–5,000).
- Cells obtained by needle washing of FNA fixed in FineFix® (Milestone Medical Technologies Inc; Kalamazoo, MI). Half sample was processed for cell-block preparation for routine diagnosis [19], the other half was stored at −80°C and used for the analysis. The quantity of cancer cells present in each needle-washing sample was at least 1,000, as inferred from the histological analysis of the corresponding cell-block.

### DNA extraction

- Cells scraped from the original cytology slides: coverslips were removed by immersion in xylene for 72 hours and the slides were rinsed in 95% ethanol three times. Cells on the slides were scraped in 1.5 ml tubes by using sterile razors. DNA was isolated using the QIAmp DNA Mini kit (Qiagen, Milano, Italy).
- Cells recovered from washing of fine-needles: samples were centrifuged at 12,000 g for 10 min to discard FineFix® and washed in PBS. DNA was isolated from the cell pellets using the QIAmp DNA Mini kit (Qiagen).
- Formalin-fixed paraffin-embedded tumor biopsies: four 10 µm paraffin sections were manually microdissected to ensure that each tumor sample contained at least 70% neoplastic cells. DNA was isolated using the QIAmp DNA FFPE tissue kit (Qiagen).

DNA was quantified and its quality assessed using NanoDrop® (Invitrogen Life Technologies; Milan, Italy) and Qubit® (Invitrogen Life Technologies) platforms according to the manifacturers' instructions.

### Deep Sequencing of Multiplex PCR Amplicons

Deep sequencing were performed using the Ion Torrent platform (Life Technologies), according to the manufacturer's specifications. Briefly, 10 ng of purified genomic DNA were used for library construction with the Ion AmpliSeq Colon and Lung Cancer Panel v1 (Life Technologies) that targets 504 mutational hotspot regions of the following 22 cancer-associated genes, in alphabetical order: *AKT1, ALK, BRAF, CTNNB1, DDR2, EGFR, ERBB2, ERBB4, FBXW7, FGFR1, FGFR2, FGFR3, KRAS, MAP2K1, MET, NOTCH1*, *NRAS*, *PIK3CA*, *PTEN*, *SMAD4*, *STK11*, *TP53*.

Emulsion PCR was performed either manually or with the OneTouch DL system (Life Technologies). The quality of the obtained library was evaluated by the Agilent® 2100 Bioanalyzer on-chip electrophoresis (Agilent Technologies; Santa Clara, CA). Sequencing was run on the Ion Torrent Personal Genome Machine™ (PGM, Life Technologies) loaded with a 316 chip as per manufacturer's protocol. Data analysis, including alignment to the hg19 human reference genome and variant calling, was done using the Torrent Suite Software v.3.2 (Life Technologies). Filtered variants were annotated using both the Ion Reporter software v1.2 (Life Techniologies) and the SnpEff software v.3.0 [20] (alignments visually verified with the Integrative Genomics Viewer; IGV v.2.1, Broad Institute [21]).

DNA from normal human lymphocytes and from the carcinoma cell line AVC1 [22] were retrieved from the ARC-NET biobank at Verona University and respectively used as negative and positive control for assessment of sensitivity.

### DNA Sanger Sequencing

To validate the mutations detected by deep sequencing, *EGFR* (exons 18, 19, 20 and 21) and *KRAS* (Exon 2) specific PCR fragments were analyzed by conventional Sanger sequencing [23]. PCR products were purified using Agencourt AMPure XP magnetic beads (Beckman Coulter) and labelled with Big Dye Terminator v3.1 (Applied Biosystems, Monza, Italy). Agencourt CleanSEQ magnetic beads (Beckman Coulter) were used for post-labeling DNA fragment purification, and sequence analysis was performed on an Applied Biosystems 3130xl Genetic Analyser.

### High resolution melting analysis

DNA was amplified for human *EGFR* (exons 19 and 21) and *KRAS* (exon 2) genes via real-time PCR, as previously described [24], in the presence of a proprietary saturating DNA dye contained in the LightCycler 480 High Resolution Melting Master (Roche Diagnostics, Milano, Italy) on the LightCycler 480 platform. A melting curve was produced using high data acquisition rates, and data were analyzed with the LightCycler 480 Gene Scanning Software Module for deletion and mutation identification.

## Results

### Patient Characteristics

The male/female ratio was 24/14 and mean age was 69±9 years (median = 68; range = 48–85). Tumor grading ranged from well (*n* = 7) to moderately (*n* = 27) or poorly differentiated (*n* = 4). In 22 cases a clinical TNM was available, 7 were Stage IIIA, 4 Stage IIIB and 11 Stage IV.

### Deep sequencing of multiplex PCR products is sensitive in mutation assessment

The sensitivity of our experimental setup was tested by progressively diluting DNA from AVC1 cancer cells with DNA from normal human lymphocytes, to obtain samples with decreasing relative tumor DNA content: 50%, 25%, 20%, 15%, 10%, 7.5%, 5%, 2.5%, 1%, and 0%. Ten ng of each dilution point were subjected to Ion AmpliSeq Colon and Lung Cancer Panel analysis v1 (Life Technologies).

A known *KRAS* mutation of the AVC1 cell line [22] was used to assess the assay sensitivity; a novel *CTNNB1* S45F mutation was also found and served to further confirm the assay sensitivity at a second genomic location. The two mutations were identified in all samples containing tumor DNA, and were absent in the sample containing only non-tumor DNA from lymphocytes (data not shown).

### Prevalence of driver genes mutations in lung adenocarcinoma cytology specimens

Ten nanograms of DNA were processed according to the manufacturers' protocol. In 36/38 (95%) samples, an adequate library for subsequent sequencing was obtained. No library amplification was observed in two scraped slides-derived samples.

**Figure 1:**
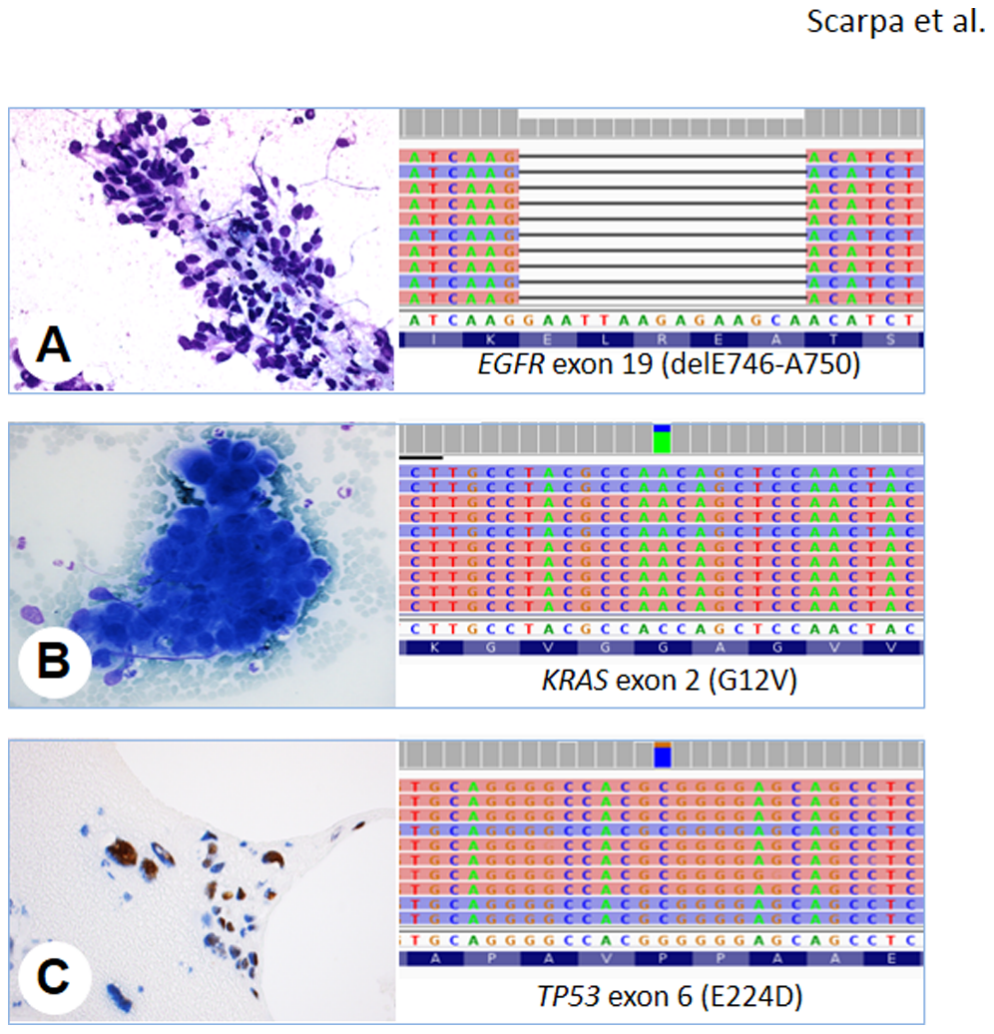
Cytological preparations from transthoracic fine needle aspiration of lung nodules and mutations identified using next generation sequencing. **A**) Case #34, a cluster of tumor cells with hyperchromatic nuclei and a high N/C ratio (Papanicolau stain, original magnification ×20); **B**) Case #29, a solid aggregate of tumor cells with dark nuclei, evident nucleoli and scarce, clear cytoplasm (Diff Quick, original magnification ×20); **C**) Case #18, section of paraffin-embedded cell block showing p53 immunostaining of cancer cells, where the nuclear accumulation of the mutated protein is evident (original magnification ×20). On the right of each sample is the representation of the reads aligned to the reference genome as provided by the Integrative Genomics Viewer (IGV v.2.1, Broad Institute) software [21].

In 24/36 (67%) samples at least one mutation was observed among the 22 lung cancer-related genes (**Table 2**, **Figure 1**). *EGFR* and *KRAS* mutations were 6/36 (16%) and 10/36 (28%), respectively. Seven mutations were identified in the *TP53* gene (18%), three in *PIK3CA* (8%), two in *BRAF* (5%), one each in *SMAD4 (3%)*, *STK11 (3%)*, and *MAP2K1* (3%). Germline variants in *MET* (T1010I) were observed in two cases and in *FGFR3* (F384L) in one case. Two cases (5%) harbored an A to T nucleotide substitution in *STK11* gene at the intronic position chr19:g.1221210.

**Table 2:** Mutations found in 24 lung adenocarcinoma cytology specimens sequenced for 22 cancer-related genes using the Ion AmpliSeq Colon and Lung Cancer Panel v1.

*Sample*	*Type*	*# cells*	*Gene mutations*										
			*EGFR*	*KRAS*	*PIK3CA*	*BRAF*	*TP53*	*PTEN*	*MET*	*SMAD4*	*FGFR3*	*STK11*	*MAP2K1*
#8	F	>100	L858R (62.9%)	-	-	-	-	-	-	-	-	-	-
#18	F	>100	delK745-A750 (64.8%)	-	-	-	E224D (70.8%)	-	-	-	-	-	-
#23	S	400	delL747-P753insS (73.2%)	-	-	-	E285K (16.3%)	-	-	-	-	-	Y130C (4.1%)
#30	S	1,000	L858R (17.3%)	-	-	G469R (2.5%)	-	-	-	-	-	-	-
#31	S	300	delE746-A750 (27.5%)	-	-	-	-	-	-	-	-	-	-
#34	S	1,000	delE746-A750 (74%)	-	-	-	-	-	-	-	-	-	-
#3	F	>100	-	G12A (40.7%)	-	-	-	-	-	Q224/stop (6.4%)	-	E70/stop (21.9%)	-
#4	F	>100	-	G12V (22.6%)	-	-	-	-	-	-	-	-	-
#9	F	>100	-	G12V (36.5%)	-	-	-	Q171stop (26.1%)	T1010I (40.8%)	-	-	chr19:g.1221210A>T (46.3%)	-
#11	F	>100	-	G12/C (8.2%)	-	-	-	-	-	-	-	-	-
#14	F	>100	-	G13C (31.5%)	-	-	-	-	-	-	-	-	-
#22	F	>100	-	G13C (48.4%)	-	-	-	-	-	-	-	-	-
#28	F	>100	-	G12D (29.6%)	-	-	-	-	-	-	-	-	-
#29	S	500	-	G12V (74.9%)	-	-	-	-	-	-	-	-	-
#32	S	750	-	G12C (43.8%)	-	-	Q165/stop (71.2%)	-	-	-	-	-	-
#35	S	250	-	G12D (30.6%)	-	-	R273L (12.8%)	-	-	-	-	-	-
#38	S	5,000	-	G12V (52.7%)	-	-	-	-	-	-	-	-	-
#1	F	>100	-	-	M1043V (6.5%)	-	G105C (5.1%)	-	-	-	F384L (55.2%)	-	-
#7	F	>100	-	-	H1047R (6.7%)	-	-	-	-	-	-	-	-
#26	S	150	-	-	D1029Y (6.0%)	D594E (83.0%)	-	-	-	-	-	-	-
#13	F	>100	-	-	-	-	Y220C (41.0%)	-	-	-	-	-	-
#17	S	250	-	-	-	-	S241Y (17.0%)	-	-	-	-	-	-
#21	F	>100	-	-	-	-	-	-	T1010I (40.3%)	-	-	-	-
#10	S	1,000	-	-	-	-	-	-	-	-	-	chr19:g.1221210A>T (24.5%)	-
**Total**			**6/36**	**10/36**	**3/36**	**2/36**	**7/36**	**1/36**	**2/36**	**1/36**	**1/36**	**3/36**	**1/36**
**%**			**15.8**	**27.8**	**7.9**	**5.3**	**18.4**	**2.6**	**5.3**	**2.6**	**2.6**	**7.9**	**2.6**

**Note**: F =  fine needle aspirate washing; S =  smear cytology.

All *EGFR* and *KRAS* mutations were confirmed at Sanger sequencing or high resolution melting analysis. *EGFR*, *KRAS* and *PIK3CA* mutations were mutually exclusive.

Nine cancers (25%) were found to have multiple driver gene alterations (**Table 2**). In these cases, significant differences were observed in the proportion of alleles affected for distinct genes, supporting the presence of intra-tumor molecular heterogeneity. For example, case #23 had 73.2% of alleles with an *EGFR* exon 19 deletion coexisting with a 16.3% of *TP53* E285K and a 4.1% of *MAP2K1* Y130C. No significant association between type and number of mutations and clinico-pathological data was observed.

### Technology reproducibility

In two FNA-washing cases (#1 and #18), two different cell sample aliquots were available. To test the Ion Torrent technology intra-sample reproducibility, 10 ng of DNA obtained from each aliquot were deep sequenced with the Ion AmpliSeq Colon and Lung Cancer Panel. In both cases the mutations identified in the first aliquot were confirmed in the second one with comparable mutation frequencies (**Table 3**).

**Table 3:** Inter-sample reproducibility as assessed in two different sample aliquots.

Sample	Gene	1^st^ aliquot	2^nd^ aliquot
	*PIK3CA*	M1043V (6.5%)	M1043V (9.0%)
#1	*TP53*	G105C (5.1%)	G105C (8.9%)
	*FGFR3*	F384L (55.2%)	F384L (55.1%)
#18	*EGFR*	delK745-A750 (64.8%)	delK745-A750 (62.2%)
	*TP53*	E224D (70.8%)	E224D (66.0%)

A total of 10 ng of DNA obtained from each aliquot were processed and sequenced.

In two cases (#8 and #21) a matched tumor biopsy, collected after cytological examination, was available and processed for deep sequencing with the Ion AmpliSeq Colon and Lung Cancer Panel. Case #8 showed an *EGFR* exon 21 L858R mutation in both samples which was also confirmed at Sanger sequencing (data not shown). In case #21, the germline variant observed in *MET* (T1010I) was confirmed in both cytological and bioptic samples, while an additional *EGFR* exon 21 L858R mutation was observed only in the biopsy sample (5.5% of analyzed codons), suggesting that the cytological sample did not contain the cancer cells harboring this mutation due to sampling variability. This mutation was confirmed at high resolution melting analysis, but not at Sanger sequencing (data not shown).

## Discussion

Subgroups of lung adenocarcinomas are characterized by specific driver molecular alterations that also represent potential therapeutic targets. Mutations in the tyrosine kinase domain of the EGFR gene are driver alterations and predictive biomarkers of response to treatment with specific inhibitors that are already in clinical practice [13], [25]. Several other driver molecular alterations have been identified in lung adenocarcinoma, including somatic mutations of *KRAS*, *BRAF*, *STK11*, *DDR2* and members of the *FGFR* family, as well as *ALK* rearrangements [10], [26], [27], [28], [29], [30], [31]. Clinical trials are ongoing in lung cancers carrying *PIK3CA*, *BRAF* or *KRAS* mutations [32], [33], [34], [35], [36]. Therefore, a comprehensive molecular characterization of lung tumors is needed for patients to benefit from novel therapeutics in either clinical practice or trials.

In addition, since some driver mutations are mutually exclusive, detection of specific molecular alterations might also predict resistance to specific drugs, as suggested for *KRAS* mutated cancers treated with EGFR tyrosine kinase inhibitors [32], [33], [34], [35], [36]. However, increasing evidence suggest that different molecular alteration may coexist in the same tumor and this might lead to acquired resistance to targeted agents. Therefore, assessment of molecular heterogeneity within the same cancer might be important in predicting the extent and duration of the response to treatment.

In order to improve the development of personalized medicine in lung adenocarcinoma, a multigene diagnostic approach, starting from a limited amount of DNA, has become mandatory in routine practice for the selection of patients most likely to benefit from targeted therapies [10], [14], [37], [38]. However, the limited diagnostic material available in most NSCLC cases is incompatible with a comprehensive molecular characterization by conventional techniques [5], [39].

In the present study, we show that targeted NGS using the Ion Torrent technology provides information about multiple genes starting from a very limited amount of DNA. In fact, in spite of a low amount of DNA input necessary for the analysis (*i.e.*, 10 ng), Ion AmpliSeq Colon and Lung Cancer Panel can simultaneously interrogate 504 hotspot mutations in 22 lung cancer-associated oncogenes and tumor suppressor genes.

The prevalence and type of mutations detected in our series of cytology samples are comparable to those reported by The National Cancer Institute Lung Cancer Mutation Consortium in 1,000 lung adenocarcinomas [40], which were: *KRAS* 25%, *EGFR* 23%, *BRAF* 3%, *PIK3CA* 3%, *MET* amplifications 2%, *ERBB2* 1%, *MAP2K1* 0.4%, and *NRAS* 0.2% [40]. *ALK* rearrangements, which are not detected by our assay, were found by FISH analysis in 6% of cases [40].

NGS analyzing the exome, i.e. the portion of genome codifying for proteins, or the entire genome has already been demonstrated to provide comprehensive molecular characterization of NSCLC [41], [42], [43], however this approach is not clinically applicable as of today. NGS technology has also recently shown to be high sensitive in EGFR single gene testing in cytology samples obtained from bronchoalveolar lavage and pleural fluid of lung adenocarcinoma patients [44]. Moreover, specific multiplex PCR assays targeting fusion genes (i.e. ALK and ROS1) are under development in the framework of the Onconetwork Consortium.

Ion AmpliSeq Colon and Lung Cancer Panel sensitivity resulted higher than that of routine molecular determinations, enabling the detection of sequence variants down to 1% allele frequency, which corresponds to 2% cancer cells in a sample. This is important in the assessment of clonal heterogeneity, i.e., the existence and quantification of multiple clones in the tumor mass [45]. This is imperative in the therapeutic management and is not achievable by conventional sequencing, which requires a sample with at least 10% tumor cells for accurate detection of mutations. In some cases, the presence of a major driver mutation coexisting with other variants displaying lower allele frequencies, as in case #23 showing *EGFR* mutation in 73% of alleles and at lower frequency in *TP53* (16%) and *MAP2K1* (4%) sustains the hypothesis of tumor molecular heterogeneity and further underlines the demand of a NGS approach to characterize the samples. In addition, case #26 may represent a good example of a patient that may be candidate to a target therapy with a *BRAF* inhibitor instead of conventional therapy as the neoplasm shows a dominant *BRAF* mutation in 83% of alleles.

The application of the Ion AmpliSeq Colon and Lung Cancer Panel in routine pathology molecular diagnostics needs validation in larger series of cases. However, its performances in detecting a wide range of genetic alterations with an extremely high sensitivity and specificity can help to assess tumor-specific therapeutic susceptibility and individual prognosis. The upcoming challenge lies in the reliable identification of an ultimate NSCLC-specific multigene panel to significantly improve the care of lung cancer patients.
